# p53 regulates enhancer accessibility and activity in response to DNA damage

**DOI:** 10.1093/nar/gkx577

**Published:** 2017-07-13

**Authors:** Scott T. Younger, John L. Rinn

**Affiliations:** 1 Department of Stem Cell and Regenerative Biology, Harvard University, Cambridge, MA 02138, USA; 2 Broad Institute of MIT and Harvard, Cambridge, MA 02142, USA; 3 Beth Israel Deaconess Medical Center, Boston, MA 02215, USA

## Abstract

The tumor suppressor p53 is a well-characterized transcription factor that can bind gene promoters and regulate target gene transcription in response to DNA damage. Recent studies, however, have revealed that p53 binding events occur predominantly within regulatory enhancer elements. The effect of p53 binding on enhancer function has not been systematically evaluated. Here, we perform a genome-scale analysis of enhancer activity from p53-bound sequences using a series of massively parallel reporter assays (MPRAs) coupled with the assay for transposase-accessible chromatin (ATAC-Seq). We find that the majority of sequences examined display p53-dependent enhancer activity during the DNA damage response. Furthermore, we observe that p53 is bound to enhancer elements in healthy fibroblasts and poised for rapid activation in response to DNA damage. Surprisingly, our analyses revealed that most p53-bound enhancers are located within regions of inaccessible chromatin. A large subset of these enhancers become accessible following DNA damage indicating that p53 regulates their activity, in part, by modulating chromatin accessibility. The recognition and activation of enhancer elements located within inaccessible chromatin may contribute to the ability of the p53 network to function across the diverse chromatin landscapes of different tissues and cell types.

## INTRODUCTION

The tumor suppressor p53 is a master regulator of the DNA damage response and a central line of defense against genomic instability (1–6). Following DNA damage p53 functions as a transcription factor and regulates the expression of a diversity of genes that influence cell fate decisions (7). Although several genome-scale analyses of the p53 network have been performed, most of these studies have focused exclusively on interactions between p53 and gene promoters (8–10). The vast majority of p53 binding sites occur outside of gene promoters and the functional impact of these sites on the DNA damage response remains unclear.

One recent analysis of the p53 network in human fibroblasts identified seven p53 binding sites that occur outside of gene promoters and harbor histone modifications consistent with regulatory enhancer elements (11). Concurrently, an independent study in *Drosophila* uncovered an additional intergenic p53 binding site that functions as an enhancer element (12). In the aforementioned studies the p53-bound enhancers were shown to regulate the expression of distant genes through mechanisms that involve chromosome looping. Subsequent reports have identified hundreds of putative p53-regulated enhancers demonstrating that enhancer regulation by p53 may be a more widespread phenomenon and an important part of the p53 network (13–15). To validate this possibility a genome-scale evaluation of enhancer activity from p53 binding sites is required.

Here, we integrate multiple functional genomics approaches and provide a systematic analysis of enhancer activity from p53 binding sites throughout the genome. We have designed and performed a series of massively parallel reporter assays (MPRAs) and quantified the regulatory capacity of p53-bound sequences during the DNA damage response. Moreover, we have incorporated the use of the assay for transposase-accessible chromatin (ATAC-Seq) and profiled the regulatory activity of p53 binding sites in their endogenous genomic contexts. Collectively, these data demonstrate that p53 modulates the activity of hundreds of enhancer elements throughout the genome.

In addition to confirming that many p53 binding sites function as enhancer elements during the DNA damage response, our analyses uncovered several previously unappreciated aspects of genome regulation by p53. We observe that p53 is bound to enhancers in healthy fibroblasts and is poised for rapid activation in response to DNA damage. Intriguingly, we find that most p53-bound enhancers reside within regions of inaccessible chromatin. The chromatin surrounding many of these enhancers becomes accessible in response to DNA damage indicating that p53 regulates their activity, in part, by modulating chromatin accessibility. Altogether, our results provide strong evidence that the regulation of enhancer accessibility and activity by p53 is an integral component of the p53 network.

## MATERIALS AND METHODS

### Cell culture

Human fetal fibroblasts GM06170 (Coriell Cell Repositories) were cultured in Dulbecco's Modified Eagle's Medium (DMEM) supplemented with 15% fetal bovine serum (Life Technologies). Fibroblasts were cultured with 500 nM doxorubicin for 6 h in MPRA experiments and 12 h in ATAC-Seq experiments to induce DNA damage.

### MPRA plasmid pool transfection

Lipofectamine 3000 (Life Technologies) was used to deliver MPRA plasmid pools into fibroblasts as per the manufacturer's instructions. Fibroblasts were plated in six-well dishes at a density of 50K cells/well. Fibroblasts were transfected with MPRA plasmid pools (1 μg/well) 24 h after plating. Culture medium was replaced 24 h after transfection with fresh medium containing 500 nM doxorubicin (Sigma) to induce DNA damage. Fibroblasts were harvested 6 h post-treatment for RNA and protein isolation.

### Western blot

Cell pellets were lysed and protein concentrations were quantified by BCA assay (Pierce). Western blots were performed on protein lysates (30 μg/well). Primary antibodies used were α-p53 (Cell Signalling Technology, 2524S) and α-GAPDH (Santa Cruz). Protein was visualized with horseradish peroxidase-conjugated protein A (Life Technologies) and Supersignal developing solution (Pierce).

### RNA isolation and quantitative PCR

RNA from treated fibroblasts was isolated using TRIzol (Life Technologies) as per the manufacturer's instructions. For each sample, 2 μg of RNA was reverse transcribed using SuperScript III Reverse Transcriptase (Life Technologies). RNA was treated with DNase I (Worthington) prior to reverse transcription. qPCR was performed on an ABI7900HT real-time PCR (Applied Biosystems) using FastStart Universal SYBR Green Master-Rox (Roche). Primers for TBP mRNA were supplied by Applied Biosystems. Primers for p21 were designed using Primer3. Only those primer sets that showed linear amplification over several orders of magnitude were used for quantification. Primers and PCR conditions are listed in Supplemental Table S8.

### Chromatin immunoprecipitation

ChIP experiments were performed as previously described (13). α-p53 (2524S), α-phospho-p53-Ser15 (9284S) and normal IgG (2729S) antibodies were supplied by Cell Signaling. Primers for p53 binding sites were designed using Primer3. Only those primer sets that showed linear amplification over several orders of magnitude were used for quantification. Primers and PCR conditions are listed in Supplemental Table S8.

### MPRA targeted sequencing libraries

MPRA targeted sequencing libraries were generated directly from 50% of each cDNA reaction using PfuUltra II Fusion HS DNA polymerase (Agilent). Libraries were size-selected using Agencourt AMPure XP beads (Beckman Coulter). Prior to sequencing, the quality and concentration of each library was assessed using the Bioanalyzer (Agilent). Primers and PCR conditions are listed in Supplemental Table S8.

### MPRA expression analysis

MPRA targeted sequencing libraries were generated such that the first 10 bases of each read corresponded to the 10-base tag used to uniquely identify individual oligonucleotides in the MPRA library. Only those sequencing reads in which the first 10 bases matched perfectly to a tag in the MPRA library and the following 26 bases matched the expected MPRA reporter sequence were used for quantification. Expression driven by each variable sequence in the library was defined by the sum of the reads mapping to each of the 10 distinct tags corresponding to the respective variable sequence. Differential expression between untreated and doxorubicin-treated fibroblasts was assessed using DESeq2 (16). For analysis of basal MPRA activity in untreated fibroblasts expression was normalized to the input MPRA vector pool.

### MPRA sequence activity contribution analysis

To determine the activity contribution of each nucleotide at each position within the variable region of the MPRA library, the sum of the activity (absolute fold-change in response to doxorubicin treatment) for each element in which base *N* appeared at position *i* was normalized by the frequency at which base *N* appeared at position *i*. Activity contributions were rescaled such that the maximum calculated activity contribution was equal to 1.

### ATAC-Seq library generation

Fibroblasts were treated with 500 nM doxorubicin for 12 h to induce DNA damage. Following treatment, ∼100K cells were resuspended in transposition reaction mix (Nextera XT DNA Library Preparation Kit, Illumina) and incubated at 37**°**C for 45 min. Transposed DNA was isolated using the MinElute PCR Purification Kit (Qiagen). ATAC-Seq libraries were generated from transposed DNA using NEBNext High-Fidelity 2X PCR Master Mix (NEB). Libraries were size-selected using Agencourt AMPure XP beads (Beckman Coulter). Prior to sequencing, the quality and concentration of each library was assessed using the Bioanalyzer (Agilent). Primers and PCR conditions are listed in Supplemental Table S8.

### ATAC-Seq analysis

ATAC-Seq reads were aligned to the human (hg19) genome using Bowtie2 (17). Aligned reads were analyzed using Scripture (Broad) to generate a catalog of potential regions with differential chromatin accessibility (18). To identify regions with significant differences in chromatin accessibility, differential read coverage between ATAC-Seq libraries from untreated and doxorubicin-treated fibroblasts was assessed with Cuffdiff2 using the Scripture output as a reference annotation (19). Analyzed chromosomal regions with <1 FPKM (fragments per kilobase of region per million fragments mapped) in untreated cells and >2 FPKM in doxorubicin-treated cells were considered ‘pioneering sites’. Regions with FPKM values <1 in untreated cells and <1.5 in doxorubicin-treated cells were considered ‘constitutively inaccessible’. Regions with <1 FPKM in untreated cells and 1.5 FPKM to 2 FPKM in doxorubicin-treated cells were considered ‘intermediate accessibility’. Regions with FPKM values >1 in both treatments were considered ‘constitutively accessible’. All visualizations represent the aggregate read coverage from three biological replicates.

### RNA sequencing and expression analysis

RNA sequencing reads were mapped to UCSC known genes and our previously described catalog of noncoding RNAs using TopHat2 with default options (20,21). Differential gene expression in response to treatment with doxorubicin was assessed using Cuffdiff2 with default options (19). For GRO-Seq, reads were aligned to the human (hg19) genome using Bowtie2 (17). Differential read coverage across genomic regions ranging from 1 kb upstream to 1 kb downstream of previously characterized p53 binding sites was assessed using Cuffdiff2.

### Transcription factor enrichment analyses

Enrichment of transcription factor (TF) binding within pioneering sites was calculated using the formula: enrichment = (s/S)/(g/G); where s = the number of TF peaks overlapping a pioneering site, S = the number of nucleotides covered by pioneering sites/the number of nucleotides covered by TF peaks, g = the number of TF peaks in the genome, and G = the number of nucleotides in the genome/the number of nucleotides covered by TF peaks. To calculate statistical significance of enrichment the labels of all TFs were randomly shuffled and the analysis was repeated 100 times. The resulting *P*-values were corrected for multiple hypothesis testing using the Bonferroni method. Significance analysis of transcription factor binding site co-occurrence within pioneering sites was calculated using the hypergeometric distribution given the number of pioneering sites overlapped by each factor under comparison and the total number of pioneering sites in the genome. Resulting *P*-values were corrected for multiple hypothesis testing using the Bonferroni method.

### Acquisition of publicly available datasets

Human transcription factor binding sites were obtained through the transcription factor ChIP-Seq Clusters Version 3 track from the UCSC Genome Browser.

## RESULTS

### Massively parallel reporter assay design

To systematically evaluate the capacity of p53-bound sequences to function as regulatory enhancer elements during the DNA damage response we designed and performed a series of massively parallel reporter assays (MPRAs). The MPRA is a high-throughput sequencing-based method for simultaneously characterizing the regulatory potential for thousands of defined DNA sequences in a single experiment (22,23). For the MPRA we designed 12,000 unique 150-mer oligonucleotides, each containing a 95 base variable region as well as a unique 10 base barcode sequence (Supplementary Table S1). The sequences of the variable regions were selected based on our previous analysis of p53 binding sites in primary human fibroblasts (13). Each of the variable regions in the MPRA oligonucleotide pool was assigned 10 independent barcode sequences such that the 12,000 oligonucleotides in the pool represent a total of 1,200 unique variable regions.

**Figure 1.**
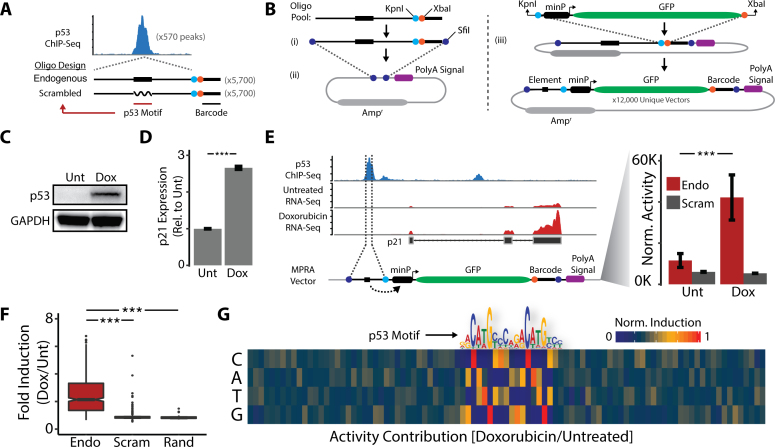
p53-bound sequences regulate proximal enhancer activity. (**A**) Schematic of MPRA oligonucleotide library design. (**B**) Schematic of Promoter-Proximal MPRA vector design and cloning. (**C**) Western blot analysis of p53 activation following doxorubicin treatment. (**D**) RT-qPCR analysis of p21 induction following doxorubicin treatment. Error bars indicate SEM (*n* = 3). *P*-values were calculated using the two-tailed unpaired Student's *t*-test with equal variances. ***P < 0.001. (**E**) MPRA analysis of enhancer activity from the p21 promoter in response to doxorubicin treatment. Error bars indicate SEM (*n* = 2). *P*-values were calculated using the Wald test. ***P < 0.001. (**F**) MPRA analysis of enhancer activity from all sequences in the MPRA oligonucleotide library in response to doxorubicin treatment. *P*-values were calculated using the two-tailed unpaired Student's *t*-test with equal variances. ***P < 0.001. (**G**) Quantitative sequence activity contribution of all MPRA oligonucleotides following doxorubicin treatment.

For the MPRA oligonucleotide pool we designed 5,700 oligonucleotides with variable regions corresponding to the genomic sequences of 570 p53 binding sites throughout the human genome (Figure 1A). In parallel, we designed an additional 5,700 oligonucleotides that were identical to the aforementioned genomic sequences with the exception that the p53 recognition motifs within the variable regions were randomly scrambled (Figure 1A). The remaining 600 oligonucleotides in the pool represented 60 variable regions comprised of random sequences that do not occur in the human genome.

To determine which of the variable regions in the MPRA oligonucleotide pool can function as enhancer elements we designed an expression vector system in which the variable regions are placed immediately upstream of a reporter gene promoter (we term this a Promoter-Proximal MPRA). In this two-step cloning approach the oligonucleotide pool is first cloned into an empty (no reporter gene) plasmid backbone (Figure 1B). The resulting vector pool is subsequently digested with restriction enzymes that separate each variable region from its associated barcode sequence. A reporter cassette comprised of a minimal promoter followed by GFP is then cloned between the variable region and the barcode sequence (Figure 1B). In the final vector pool each GFP reporter gene contains the unique barcode sequence that allows the reporter transcript to be associated with the upstream variable region modulating its expression.

### p53-bound sequences regulate proximal enhancer activity

To measure the regulatory activity of variable regions within the MPRA oligonucleotide pool during the DNA damage response the Promoter–Proximal MPRA vector pool was transfected into GM06170 primary human fibroblasts. Transfected fibroblasts were then cultured in the presence of 500 nM doxorubicin for 6 hours to activate the p53 response to DNA damage. Treatment with doxorubicin resulted in a robust increase in the expression of p53 protein (Figure 1C). Furthermore, doxorubicin treatment significantly activated the expression of p21, a well-characterized transcriptional target of p53 (Figure 1D). These results demonstrate that the p53 response to DNA damage remains functional in cells harboring MPRA expression vectors.

In order to monitor expression levels from the Promoter-Proximal MPRA vector pool we next performed targeted RNA-Seq. We generated sequencing libraries by amplifying the region of the GFP reporter transcripts containing the unique barcode sequences. The representation of each barcode within the sequencing library provides a digital readout for the regulatory activity of the upstream genomic element. As a positive control we evaluated the regulatory activity of sequences within the MPRA pool corresponding to the p53 binding site within the p21 promoter. Treatment with doxorubicin resulted in a significant increase in expression from Promoter-Proximal MPRA vectors containing the endogenous sequence of this p53 binding site (Figure 1E). In contrast, MPRA vectors containing the analogous sequence in which the p53 recognition motif was scrambled were not affected by doxorubicin treatment (Figure 1E).

Interestingly, we observed that expression from Promoter-Proximal MPRA vectors containing the endogenous p21 promoter sequence was higher than their scrambled counterparts even in untreated fibroblasts (Figure 1E). This observation suggests that a basal pool of p53 (undetectable by Western blot) may be bound to these sequences in the absence of DNA damage. To test this hypothesis we evaluated p53 binding at the p21 promoter in fibroblasts cultured in the presence or absence of doxorubicin using chromatin immunoprecipitation followed by quantitative PCR (ChIP-qPCR). We detected p53 occupancy at the p21 promoter in both untreated and doxorubicin treated cells (Supplementary Figure S1A). Doxorubicin treatment had no effect on the level of p53 binding (Supplementary Figure S1A). However, we did observe a significant increase in the level of activated p53 (phospho-p53-Ser15) in response to DNA damage (Supplementary Figure S1B). These findings indicate that p53 is bound to the p21 promoter in healthy cells and poised for activation in response to DNA damage.

We next evaluated the expression from all of the sequences represented within the Promoter-Proximal MPRA vector pool. We found that the vast majority of sequences corresponding to p53 binding sites display increased regulatory activity in response to DNA damage (Figure 1F, Supplementary Table S2). This regulatory activity was absent from Promoter-Proximal MPRA vectors containing scrambled p53 recognition motifs (Figure 1F, Supplementary Table S2). Moreover, the activity of vectors containing scrambled p53 recognition motifs was indistinguishable from vectors containing random sequences (Figure 1F). Consistent with our observations at the p21 promoter, we found that expression from Promoter-Proximal MPRA vectors corresponding to the endogenous sequences of p53 binding sites was significantly greater than the expression from their scrambled counterparts in untreated cells (Supplementary Figure S1C).

To further understand the sequence properties of p53-bound regions that drive regulatory activity we calculated the sequence activity contribution of each nucleotide in each position of the MPRA oligonucleotide pool. Our analysis identified the p53 recognition motif as the primary determinant of regulatory activity (Figure 1G). We searched for additional sequence motifs that might contribute to regulatory activity using Multiple Em for Motif Elicitation (MEME) but were unable to detect enrichment of any sequences aside from the p53 recognition motif (24). Altogether, the results from the Promoter–Proximal MPRA indicate that p53-bound sequences can function as enhancer elements when located proximal to transcription start sites.

### p53-bound sequences regulate distal enhancer activity

**Figure 2.**
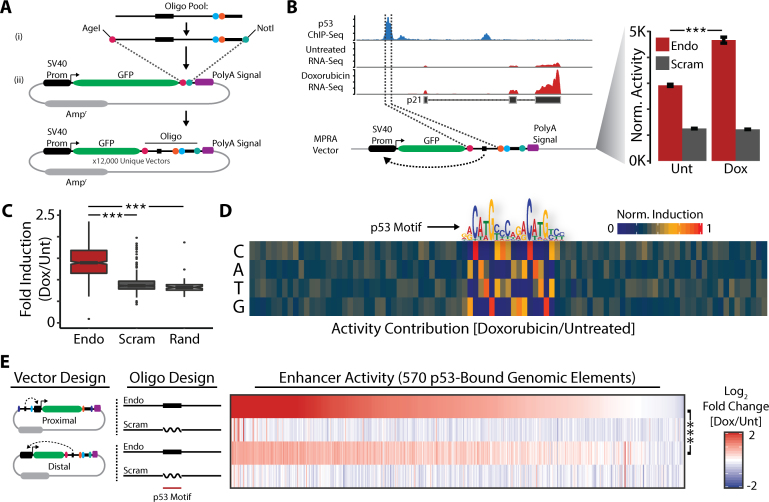
p53-bound sequences regulate distal enhancer activity. (**A**) Schematic of Promoter–Distal MPRA vector design and cloning. (**B**) MPRA analysis of enhancer activity from the p21 promoter in response to doxorubicin treatment. Error bars indicate SEM (*n* = 2). *P*-values were calculated using the Wald test. ***P < 0.001. (**C**) MPRA analysis of enhancer activity from all sequences in the MPRA oligonucleotide library in response to doxorubicin treatment. *P*-values were calculated using the two-tailed unpaired Student's *t*-test with equal variances. ***P < 0.001. (**D**) Quantitative sequence activity contribution of all MPRA oligonucleotides following doxorubicin treatment. (**E**) Heatmap of enhancer activity in Promoter–Proximal and Promoter–Distal MPRA approaches. *P*-values were calculated using the Wilcoxon signed-rank test. ***P < 0.001.

Enhancer elements can be separated from the genes that they regulate by varying genomic distances. To evaluate the ability of p53-bound sequences to function as enhancers when located more distant from transcription start sites we designed a Promoter–Distal MPRA vector pool. In this design the MPRA oligonucleotide pool is cloned ∼800 nt downstream of the transcription start site of a GFP reporter gene (Figure 2A). As a result, the sequences of the MPRA oligonucleotides are expressed within the GFP reporter transcripts and their regulatory activity can be quantified directly using targeted RNA-Seq.

The Promoter-Distal MPRA vector pool was transfected into GM06170 primary human fibroblasts, which were subsequently cultured in the presence of 500 nM doxorubicin for 6 h to activate the p53 response to DNA damage. We then performed targeted RNA-Seq to monitor expression from the Promoter-Distal MPRA vectors. As a positive control for enhancer activity we again focused on sequences within the MPRA pool corresponding to the p53 binding site within the p21 promoter. Expression from Promoter–Distal MPRA vectors containing the endogenous sequence of this p53 binding site was significantly increased in response to doxorubicin treatment, although not as robustly as with the Promoter–Proximal MPRA (Figure 2B). Promoter–Distal MPRA vectors containing the analogous scrambled p53 recognition motif sequences were unaffected (Figure 2B). We again observed far greater regulatory activity from Promoter–Distal MPRA vectors containing the endogenous p21 promoter sequence relative to their scrambled counterparts in untreated fibroblasts (Figure 2B).

We next monitored the regulatory activity from all of the sequences represented within the Promoter–Distal MPRA vector pool. We observed that most of the sequences corresponding to p53 binding sites display increased enhancer activity in response to DNA damage (Figure 2C, Supplementary Table S3). This increase was absent from MPRA vectors containing scrambled p53 recognition motifs (Figure 2C, Supplementary Table S3). To search for sequence properties of p53-bound regions responsible for distal enhancer regulation we calculated the sequence activity contribution of the MPRA oligonucleotide pool. Once again, the only determinant of regulatory activity we were able to distinguish was the p53 recognition motif (Figure 2D). These results indicate that p53-bound sequences can function as enhancer elements when located distant from transcription start sites. As with the Promoter-Proximal MPRA, we found that expression from Promoter–Distal MPRA vectors corresponding to the endogenous sequences of p53 binding sites was significantly greater than the expression from their scrambled counterparts in untreated fibroblasts (Supplementary Figure S1D). This observation supports a general model whereby p53 is bound to recognition motifs in healthy cells and is poised for activation in response to DNA damage.

To further understand how the distance between a p53-bound sequence and a reporter gene promoter affects enhancer activity we compared the results from the Promoter–Proximal and Promoter–Distal MPRAs. In general, we found that the magnitude of enhancer activation in response to doxorubicin treatment was less pronounced using the Promoter–Distal MPRA approach (Figure 2E). However, we did observe a significant correlation between the enhancer activities of the sequences corresponding to p53 binding sites across approaches (Figure 2E). These data indicate that the ability of p53-bound sequences to enhance gene expression is independent from their distances to transcription start sites.

### p53 is bound to enhancers located within inaccessible chromatin

Having established that p53-bound sequences have the capacity to function as enhancer elements using MPRA enhancer screens, a transient reporter approach, we next characterized the regulatory potential of endogenous p53 binding sites using the assay for transposase-accessible chromatin using sequencing (ATAC-Seq). Briefly, ATAC-Seq utilizes transposon integration in live cells as a method for assaying chromatin accessibility (25). Following incubation with transposase, the sequence of the transposon can be used to generate high-throughput sequencing libraries that permit the identification of transposition sites throughout the genome. Chromatin accessibility can in turn be used as a proxy for the identification of active regulatory elements.

**Figure 3.**
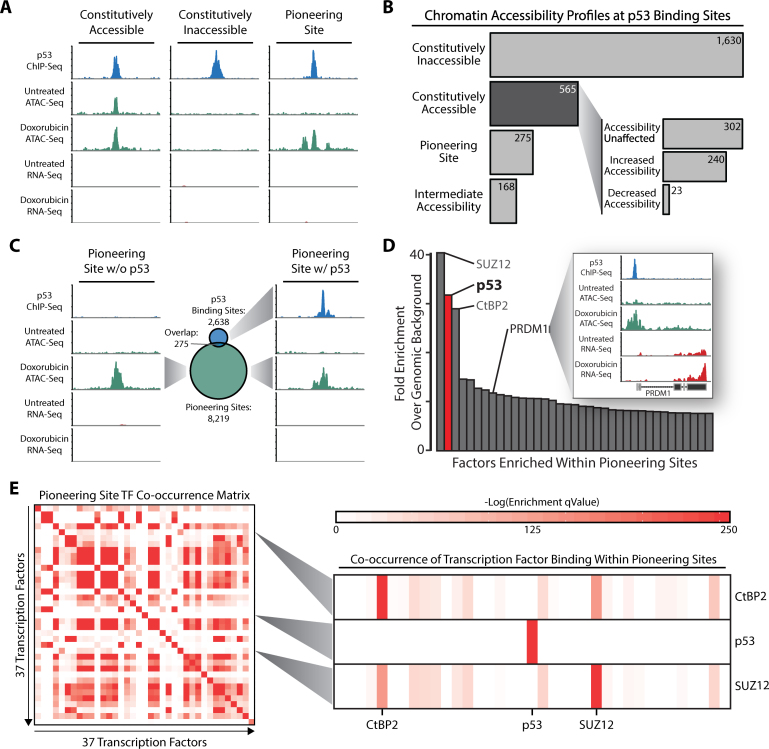
p53 regulates enhancer accessibility in response to DNA damage. (**A**) Examples of p53 binding sites within constitutively accessible chromatin, constitutively inaccessible chromatin, and pioneering sites. (**B**) Distribution of p53 binding sites within constitutively accessible chromatin, constitutively inaccessible chromatin, and pioneering sites. (**C**) Overlap of p53 binding sites and genome-wide pioneering sites. (**D**) Enrichment analysis of transcription factor binding sites within pioneering sites. (**E**) Analysis of transcription factor binding site co-occurrence significance within pioneering sites.

We cultured GM06170 fibroblasts in the presence of 500 nM doxorubicin for 12 h to activate the p53 response to DNA damage and performed ATAC-Seq. Across three biological replicates we achieved an average sequencing depth of 42 million mapped reads per sample in untreated fibroblasts and 62 million mapped reads per sample in doxorubicin-treated fibroblasts. In addition, <10% of the mapped reads in each sample aligned to mitochondrial DNA, a common contaminant in many ATAC-Seq experiments. Our analyses identified three distinct classes of chromatin accessibility profiles surrounding p53 binding sites (Figure 3A). We found that 21% (565/2,638) of binding sites are accessible prior to doxorubicin treatment. These accessible regions, which we termed ‘constitutively accessible’, represent regulatory elements that are active in healthy fibroblasts. Of these constitutively accessible sites 43% (240/565) displayed a >2-fold increase in accessibility following DNA damage suggesting that p53 further increases the activity of these elements (Figure 3B). We found that 53% (302/565) of constitutively accessible binding sites exhibited no changes in accessibility while the remaining 4% (23/565) of these sites had a >2-fold decrease in accessibility following doxorubicin treatment (Figure 3B).

Surprisingly, we found that 62% (1,630/2,638) of p53 binding sites occur within regions of the genome that are inaccessible in both untreated and doxorubicin-treated fibroblasts (Figure 3A and B). These sites, which we termed ‘constitutively inaccessible’, demonstrate that p53 is bound to response elements even when located within inaccessible chromatin. The ability to bind to sites within inaccessible chromatin is a distinguishing property of a small class of transcription factors known as pioneering factors. Pioneering factors bind to response elements within condensed chromatin and can either directly or indirectly promote increases in accessibility that permits the subsequent binding of additional regulatory factors, a process we refer to hereafter as ‘pioneering activity’ (26). While most p53 binding sites remain inaccessible in both untreated and doxorubicin-treated cells, we hypothesized that p53 may function as a pioneering factor at a subset of binding sites during the DNA damage response. Indeed, we found that chromatin surrounding 10% (275/2,638) of p53 binding sites shifts from inaccessible to accessible in response to DNA damage (Figure 3A and B). These regions, which we termed ‘pioneering sites’, suggest that p53 has the capacity to function as a pioneering factor and modulate changes in chromatin accessibility in response to DNA damage. The remaining 168 p53 binding sites that were not classified in one of the previously described categories were termed as ‘intermediate accessibility’. Many of these regions resembled pioneering sites but did not meet the stringent criteria used for classification in our ATAC-Seq analyses.

### p53 regulates enhancer accessibility in response to DNA damage

To further understand how pioneering activity at p53 binding sites relates to pioneering activity across the genome we performed a global analysis of DNA damage-induced pioneering sites. We identified 8,219 regions in the genome that displayed clear signs of pioneering activity, 275 of which are bound by p53 (Figure 3C). Because relatively few pioneering sites are bound by p53 we next searched for additional transcription factors that might contribute to the changes in chromatin accessibility at these regions. Using publicly available ChIP-Seq data sets collected from a variety of cell types we found that binding sites for 37 factors are significantly enriched within pioneering sites (Figure 3D). Despite the relatively low fraction of p53 binding events that occur within pioneering sites (275/8,219), p53 was among the most enriched factors within these regions relative to genomic background. The only factors enriched at levels comparable to p53 were SUZ12 and CtBP2. Both of these factors have known repressive functions and their enrichment at pioneering sites likely results from a role in keeping these genomic regions silent in the cell type from which their respective ChIP-Seq data were generated. Interestingly, we found that two transcription factors enriched within pioneering sites (PRDM1 and ATF3) are both direct transcriptional targets of p53 (Figure 3D). This observation suggests that, in addition to driving changes in chromatin accessibility surrounding its own binding sites, p53 induces the expression of genes that further reshape the chromatin landscape of the genome.

To characterize potential interactions between transcription factors bound to pioneering sites we next evaluated transcription factor binding site co-occurrence between all factors that are significantly enriched within pioneering sites. For 36 of the 37 factors analyzed we observed significant co-occurrence with two or more additional factors at pioneering sites (Figure 3E). Although none of the factors included in our analysis have similar consensus motifs, many of them (i.e. SUZ12 and CtBP2) shared similar co-occurrence patterns. In contrast, p53 was the only factor that exhibited no significant co-occurrence with any other factors. This indicates that pioneering activity by p53 does not require the presence of additional factors. Moreover, these p53-regulated enhancers appear to be controlled exclusively by p53 and do not crosstalk with other regulatory networks. Collectively, these results demonstrate that p53 regulates chromatin accessibility at enhancer elements during the DNA damage response.

To confirm that p53 has the capacity to function as a pioneering factor we used existing ATAC-Seq datasets to profile changes in chromatin accessibility at p53 binding sites in IMR90 fibroblasts treated with nutlin (15). Importantly, nutlin and doxorubicin increase p53 expression through distinct mechanisms. Doxorubicin treatment results in DNA double-strand breaks and leads to activation of the p53-mediated DNA damage response. In contrast, nutlin inhibits Mdm2-mediated degradation of p53 resulting in increased p53 protein expression (27). Using our ATAC-Seq analysis pipeline we found that chromatin accessibility profiles at p53 binding sites were similar across cell lines and treatments. For example, regions corresponding to constitutively inaccessible and pioneering sites in GM06170 fibroblasts displayed significantly lower ATAC-Seq read coverage in DMSO-treated IMR90 fibroblasts when compared to regions corresponding to constitutively accessible sites (Supplementary Figure S2A). Consistent with our findings in doxorubicin-treated fibroblasts, treatment with nutlin resulted in significant increases in chromatin accessibility at regions corresponding to pioneering sites (Supplementary Figure S2B). Examples of chromatin accessibility profiles at p53 binding sites are shown in Supplementary Figure S2C. These observations further support a direct role for p53 in modulating chromatin accessibility.

### Pioneering activity by p53 uncovers a potent class of enhancers

**Figure 4.**
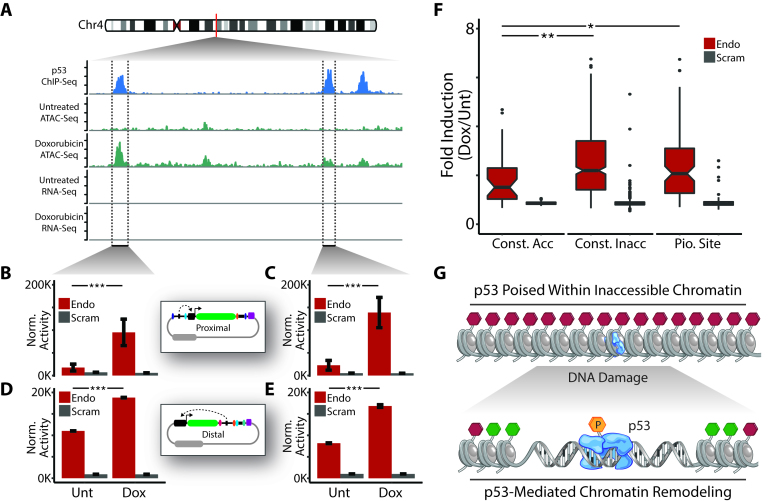
Pioneering activity by p53 uncovers a potent class of enhancers. (**A**) Examples of p53 binding at pioneering sites. (**B**–**E**) MPRA analysis of enhancer activity from pioneering sites in response to doxorubicin treatment using (B, C) Promoter–Proximal and (D, E) Promoter–Distal MPRA methods. Error bars indicate SEM (*n* = 2). *P*-values were calculated using the Wald test. ***P < 0.001. (**F**) Promoter–Proximal MPRA analysis of enhancer activity from all sequences in the MPRA oligonucleotide library (categorized by chromatin accessibility) in response to doxorubicin treatment. *P*-values were calculated using the two-tailed unpaired Student's *t*-test with equal variances. **P < 0.01, *P < 0.05. (**G**) Model for mechanism of enhancer regulation by p53. Hexagons represent putative histone modifications (red = repressive, green = active).

To characterize the relationship between chromatin accessibility and regulatory activity in response to DNA damage we integrated our MPRA enhancer screens with ATAC-Seq profiles. Of the 570 genomic regions represented in our MPRA library 34 corresponded to regions of constitutively accessible chromatin, 445 corresponded to regions of constitutively inaccessible chromatin, and 91 corresponded to pioneering sites. An example of three proximal pioneering sites bound by p53 is shown in Figure 4A. Promoter–Proximal MPRA vectors corresponding to two of these sites (the third site was not represented in our MPRA oligonucleotide pool) revealed that these sequences have significant regulatory potential (Figure 4B and C). Promoter-Distal MPRA vectors corresponding to these sites confirmed that these sequences also regulate gene expression from distal sites, further indicating that they are functional enhancer elements (Figure 4D and E). As seen previously, analogous MPRA vectors containing scrambled p53 motifs were not responsive to DNA damage (Figure 4B–E).

We then compared chromatin accessibility profiles with the activity of endogenous p53 binding sites by evaluating expression changes for nearby genes in response to DNA damage. Specifically, we incorporated our previously described analysis of differential gene expression in response to treatment with doxorubicin and compared expression changes for genes with transcription start sites that occur within 2 kb (upstream or downstream) of p53 binding sites (Supplementary Table S4) (13). Consistent with our chromatin accessibility profiles, we found that genes located near constitutively accessible and pioneering sites displayed significantly greater levels of induction in response to doxorubicin treatment as compared to genes located near constitutively inaccessible sites (Supplementary Figure S3A). Furthermore, induction of genes near pioneering sites was significantly greater than that of genes located near constitutively accessible sites (Supplementary Figure S3A). We also found that genes located near p53-bound pioneering sites display lower basal expression levels than those located near constitutively accessible sites (Supplementary Figure S3B). Expression of genes near constitutively inaccessible sites was similar to those near constitutively accessible sites indicating that genes in these sets are likely regulated by factors independent of p53 (Supplementary Figure S3B).

As an alternative method for monitoring endogenous enhancer activity we used existing datasets to profile enhancer RNA (eRNA) production at p53 binding sites. Briefly, eRNAs are noncoding RNA transcripts derived from enhancer elements and their expression is positively correlated with enhancer activity (28). A genome-wide map of eRNA production in response to p53 stabilization has recently been reported (14). While this dataset was generated using cancer cells treated with nutlin as opposed to normal fibroblasts treated with doxorubicin, we reasoned that general properties of p53 activity might be preserved across studies.

To evaluate basal enhancer activity we compared eRNA expression levels in DMSO-treated cells at regions corresponding to p53 binding sites profiled in our study (Supplementary Table S5). We found that regions corresponding to constitutively accessible p53 binding sites exhibit significantly higher basal eRNA expression levels than regions corresponding to constitutively inaccessible and pioneering sites (Supplementary Figure S4A). Stabilization of p53 resulted in significant induction of eRNA expression at p53-bound pioneering sites (Supplementary Figure S4B). In contrast, eRNA expression at the majority of constitutively accessible and constitutively inaccessible sites was not impacted by p53 stabilization (Supplementary Figure S4B). Examples of eRNA production at p53 binding sites are shown in Supplementary Figure S4C. These observations indicate that p53-bound pioneering sites represent enhancer elements that are inactive under normal conditions, but are poised for activation in response to p53 stabilization/activation.

Our previous analyses of both Promoter-Proximal and Promoter-Distal MPRA approaches, and more specifically at the p21 promoter, revealed that p53 is bound to response elements in untreated cells (Supplementary Figure S1A–D). To determine if this generalization applies to pioneering sites we performed a targeted analysis on an enhancer located on chromosome 1 (Supplementary Figure S5A). Promoter–Proximal and Promoter–Distal MPRA experiments confirmed that this region functions as an enhancer element (Supplementary Figure S5B and C). Using ChIP-qPCR we were able to detect p53 occupancy on the endogenous enhancer in both untreated and doxorubicin-treated fibroblasts (Supplementary Figure S5D). In contrast, there was a significant increase in the level of activated p53 (phospho-p53-Ser15) coinciding with the observed increase in chromatin accessibility at this site (Supplementary Figure S5E). These findings demonstrate that p53 is bound to enhancers within inaccessible chromatin and poised for activation in response to DNA damage.

Finally, we used our MPRA enhancer screen data to compare the regulatory potential of p53 binding sites based on their endogenous chromatin accessibility profiles. We found that Promoter-Proximal MPRA vectors containing sequences located at pioneering sites display significantly greater induction in response to DNA damage than those containing sequences located within regions of constitutively accessible chromatin (Figure 4F, Supplementary Table S6). Interestingly, Promoter-Proximal MPRA vectors corresponding to sequences located within constitutively inaccessible chromatin were induced at levels comparable to those containing sequences located at pioneering sites (Figure 4F). We observed similar results when comparing chromatin accessibility profiles with activity from Promoter-Distal MPRA vectors (Supplementary Table S7). Altogether, our results demonstrate that p53 is poised on a potent class of enhancers that are silenced within inaccessible chromatin but primed for activation in response to DNA damage.

## DISCUSSION

The regulation of gene expression by p53 has been studied extensively in the context of binding events that occur within gene promoters. Several recent studies, however, have found that p53 binding sites occur predominantly within regulatory enhancer elements (11,13,14). The ability of p53-bound sequences to function as enhancer elements has not been thoroughly explored. Here, we have utilized several recently described approaches in functional genomics to characterize enhancer activity from p53-bound sequences throughout the genome. We have designed and performed a series of MPRA enhancer screens and experimentally validated hundreds of p53-regulated enhancers. Moreover, we have incorporated the use of ATAC-Seq and uncovered a previously unappreciated role for p53 in the modulation of chromatin accessibility at enhancer elements during the DNA damage response.

In our MPRA enhancer screens we found that the vast majority of the genomic sequences that are bound by p53 display some level of enhancer induction in response to DNA damage. This enhancer activity is preserved regardless of the distance between the p53-bound sequence and the transcription start site of the reporter gene being regulated. While we did observe differences in the degree of enhancer activity between the many sequences we investigated, we were unable to identify any specific sequence features that explain these variations. In fact, the only distinguishable sequence property associated with enhancer activity during the DNA damage response was the presence of a p53 recognition motif. In the absence of a p53 recognition motif responsiveness to DNA damage was completely ablated, further demonstrating that p53 is the sole factor that modulates enhancer activity. Supporting our observations, an independent report published during the course of our study described the use of multiplexed reporter assays to profile enhancer activity from p53-bound regions and identified a large class of enhancers that are characterized by single p53 binding sites (29).

Interestingly, our enhancer screen revealed that p53 is poised on response elements prior to the induction of DNA damage. We confirmed at two independent endogenous loci (one promoter and one enhancer) that p53 is indeed bound prior to the induction of DNA damage. Previous reports have shown that p53 binds to a specific subset of gene promoters and recruits components of the transcription initiation complex in healthy cells (14,30,31). Our findings extend this property of p53 to regulatory enhancer elements. Furthermore, we demonstrate that the presence of poised p53 is sufficient to transform bound sequences into functional enhancer elements that are responsive to DNA damage.

While our MPRA enhancer screens succeeded in uncovering novel aspects of p53 biology, there were also some clear distinctions between the enhancer activities from MPRA expression vectors and endogenous p53-bound sequences. Specifically, we observed that many sequences in our MPRA oligonucleotide pool that exhibited significant enhancer induction in response to DNA damage actually corresponded to genomic regions located within constitutively inaccessible, thus inactive, chromatin. These differences demonstrate that the MPRA is a powerful tool for evaluating the functional potential of DNA sequences, but the actual regulatory capacity of an endogenous genomic sequence is ultimately dictated by the surrounding chromatin landscape. These are important considerations to take into account during the design and interpretation of MPRA-based approaches.

Perhaps the most striking discovery in our study was that p53 appears to function as a pioneering factor, binding to response elements located within inaccessible chromatin and modulating changes in chromatin accessibility in response to DNA damage. In contrast to previous reports demonstrating that p53 can bind to genomic regions with high nucleosome occupancy in response to DNA damage, we observe that p53 is stably bound within inaccessible chromatin in healthy cells (15,32). Whether p53 binds to these regions while chromatin is inaccessible, as opposed to binding during periods of transient accessibility, remains to be determined. Intriguingly, we find that pioneering activity only occurs at a subset of p53-bound enhancers suggesting that p53 binding is not sufficient for chromatin remodeling. However, we were unable to identify any specific sequence features or binding sites for regulatory co-factors that were predictive of pioneering activity.

We propose that p53 is poised within inaccessible chromatin in an inactive state and that p53 activation (via post-translational modification) in response to DNA damage promotes chromatin remodeling (Figure 4G). In this model pioneering activity would be governed by the activation status of p53 as opposed to just the binding status. The specific loci where pioneering activity occurs may be determined by the physical proximity of p53-modifying enzymes within the nucleus. Alternatively, p53-modifying enzymes may possess inherent selectivity towards p53 molecules that are bound to specific sites in the genome.

Finally, we present several lines of evidence demonstrating that p53 binding sites associated with pioneering activity comprise a highly potent class of p53-regulated enhancers. We find that genes located near p53-bound pioneering sites display significantly higher levels of induction in response to DNA damage as compared to genes near constitutively accessible or constitutively inaccessible binding sites. Furthermore, we observe significant increases in eRNA expression from p53-bound pioneering sites following p53 stabilization. Lastly, our MPRA screens revealed that the sequences of p53-bound pioneering sites display significantly greater enhancer activity during the DNA damage response as compared to the sequences of constitutively accessible p53 binding sites.

The potent enhancer activity from p53-bound pioneering sites could not be attributed to the presence of binding sites for any specific regulatory co-factors or other sequence properties. Rather, we hypothesize that constitutively accessible genomic regions contain binding sites for a large array of regulatory factors and that p53 must compete with all of these factors to modulate enhancer activity. Conversely, sequences located within inaccessible chromatin are less likely to contain response elements for as many regulatory factors. Therefore, the elevated enhancer activity from sequences located within pioneering sites may result from the lack of regulatory competition with other factors as opposed to the presence of any single co-factor. This regulatory competition would also explain the overall variation in the enhancer activity we observed in our MPRA enhancer screens.

In conclusion, our findings have shown that p53 binding sites throughout the genome can function as DNA damage-responsive enhancer elements. Moreover, we have uncovered a previously unappreciated role for p53 in the modulation of chromatin accessibility at enhancers during the DNA damage response. The ability to bind and activate regulatory elements located within inaccessible chromatin may explain how p53 can effectively mediate cellular stress responses across a wide variety of tissues and cell types that harbor diverse chromatin landscapes.

## ACCESSION NUMBERS

The Gene Expression Omnibus accession number for the MPRA and ATAC-Seq data reported in this paper is GSE83780.

## Supplementary Material

Supplementary Data
