# Supplementary material for: p53 regulates enhancer accessibility and activity in response to DNA damage
Source: Nucleic Acids Res. 2017 Jul 13;45(17):9889–900. doi: 10.1093/nar/gkx577 (PMC5622327; doi:10.1093/nar/gkx577)
Supplement: Supplementary Data [file gkx577_supp.zip › Younger_Supplemental_Figure_Legends.docx]

**Supplemental Figure Legends**

**Supplemental Figure S1. p53 is poised on recognition motifs in healthy fibroblasts**

(A and B) ChIP-qPCR analysis of (A) p53 and (B) phospho-p53 occupancy at the p21 promoter following doxorubicin treatment. Error bars indicate SEM (n = 3). P-values were calculated using the two-tailed unpaired Student’s t-test with equal variances. (***) P<0.001. (C and D) MPRA analysis of basal enhancer activity from all sequences in the MPRA oligonucleotide library in untreated cells using (C) Promoter-Proximal and (D) Promoter-Distal MPRA methods. P-values were calculated using the two-tailed unpaired Student’s t-test with equal variances. (***) P<0.001.

**Supplemental Figure S2. p53 regulates chromatin accessibility in response to nutlin treatment**

(A) Basal ATAC-Seq read coverage at p53 binding sites in DMSO-treated IMR90 fibroblasts (categorized by chromatin accessibility in GM06170 fibroblasts). P-values were calculated using the two-tailed unpaired Student’s t-test with equal variances. (***) P<0.001. (B) Differential ATAC-Seq read coverage at p53 binding sites in nutlin-treated IMR90 fibroblasts (categorized by chromatin accessibility in GM06170 fibroblasts). P-values were calculated using the two-tailed unpaired Student’s t-test with equal variances. (***) P<0.001. (C) Examples of chromatin accessibility profiles at p53 binding sites within constitutively inaccessible chromatin, constitutively accessible chromatin, and pioneering sites in IMR90 fibroblasts.

**Supplemental Figure S3. Pioneering activity at p53-bound enhancers is associated with gene activation**

(A) Differential expression of genes located near p53 binding sites (categorized by chromatin accessibility) in response to doxorubicin treatment. P-values were calculated using the two-tailed unpaired Student’s t-test with equal variances. (**) P<0.01, (*) P<0.05. (B) Basal expression of genes located near p53 binding sites (categorized by chromatin accessibility). P-values were calculated using the two-tailed unpaired Student’s t-test with equal variances. (***) P<0.001 , (**) P<0.01.

**Supplemental Figure S4. Pioneering activity at p53-bound enhancers is associated with enhancer activation**

(A) Basal eRNA expression at p53 binding sites in untreated fibroblasts (categorized by chromatin accessibility). P-values were calculated using the two-tailed unpaired Student’s t-test with equal variances. (***) P<0.001, (*) P<0.05. (B) Differential eRNA expression at p53 binding sites (categorized by chromatin accessibility) in response to doxorubicin treatment. P-values were calculated using the two-tailed unpaired Student’s t-test with equal variances. (***) P<0.001, (**) P<0.01, (*) P<0.05. (C) Examples of eRNA expression from p53 binding sites within constitutively inaccessible chromatin, constitutively accessible chromatin, and pioneering sites.

**Supplemental Figure S5. p53 is poised on enhancers within inaccessible chromatin**

(A) Example of p53 binding at a pioneering site. (B) Promoter-Proximal MPRA corresponding to selected enhancer locus. (C) Promoter-Distal MPRA corresponding to selected enhancer locus. (D) ChIP-qPCR analysis of p53 occupancy at selected enhancer locus in response to DNA damage. Error bars indicate SEM (n = 3). (E) ChIP-qPCR analysis of phospho-p53 occupancy at selected enhancer locus in response to DNA damage. Error bars indicate SEM (n = 3). P-values were calculated using the two-tailed unpaired Student’s t-test with equal variances. (**) P<0.01.

**Supplemental Table S1. Sequences of MPRA oligonucleotide pool**

**Supplemental Table S2. Promoter-Proximal MPRA results**

**Supplemental Table S3. Promoter-Distal MPRA results**

**Supplemental Table S4. p53 binding sites (chromatin accessibility and differential gene expression)**

**Supplemental Table S5. p53 binding sites (chromatin accessibility and differential enhancer RNA expression)**

**Supplemental Table S6. p53 binding sites (chromatin accessibility and Promoter-Proximal MPRA expression)**

**Supplemental Table S7. p53 binding sites (chromatin accessibility and Promoter-Distal MPRA expression)**

**Supplemental Table S8. PCR primers and conditions**
