# Supplementary material for: p53 regulates enhancer accessibility and activity in response to DNA damage
Source: Nucleic Acids Res. 2017 Jul 13;45(17):9889–900. doi: 10.1093/nar/gkx577 (PMC5622327; doi:10.1093/nar/gkx577)
Supplement: Supplementary Data [file gkx577_supp.zip › Younger_Supplemental_Methods.docx]

**Supplemental Materials and Methods**

**Oligonucleotide library design**

We designed 150-mer oligonucleotides containing: (a) a universal primer site, (b) a 95-base variable sequence, (c) recognition sequences for KpnI and XbaI restriction enzymes, (d) a 10-base tag sequence, and (e) a second universal primer site. Variable sequences in the library were designed based on p53 binding sites we previously identified by ChIP-Seq. Binding sites were prioritized by depth of read coverage in ChIP-Seq experiments and filtered by the presence of a p53 consensus motif (specifically [A/G]C[A/T][A/T]G[C/T][C/T][2–4 Spacer Nucleotides][G/A][G/A]C[A/T][A/T]G[T/C]). We extracted 95-base sequences (centered around the detected p53 consensus motif) and further screened for those that did not interfere with our subsequent cloning strategy. Specifically, we selected the top 570 p53-bound genomic sequences that lacked recognition sites for the restriction enzymes SfiI, KpnI, and XbaI. In addition, we designed 570 analogous sequences corresponding to the selected p53 binding sites in which the p53 consensus motif was randomly scrambled (retaining nucleotide content), ensuring that the scrambled sequence did not give rise to recognition sites for SfiI, KpnI, XbaI, or p53. In parallel, we designed 60 fully randomized 95-base sequences that: (a) lacked recognition sites for the restriction enzymes SfiI, KpnI, and XbaI, (b) lacked a p53 consensus motif, and (c) did not align to the human genome. Each of the 1,200 previously described 95-base variable sequences was represented in the oligonucleotide library with 10 distinct 10-base tag sequences, totaling 12,000 unique oligonucleotide sequences in the final library. Tag sequences were designed such that each tag: (a) contained all 4 bases, (b) did not contain regions with more than 4 consecutive identical nucleotides, (c) did not contain recognition sequences for XbaI or KpnI restriction enzymes, (d) did not contain known human miRNA seed sequences (obtained from miRBaseV21), and (e) was represented only once in the oligonucleotide library. Sequences of the oligonucleotide library are listed in Supplemental Table S1.

**Promoter-Proximal Oligonucleotide library amplification**

The oligonucleotide library was amplified by PCR with Q5 High-Fidelity DNA Polymerase (NEB) and primers targeting the universal primer sequences within the oligonucleotides. To preserve library complexity PCR was performed using the Micellula DNA Emulsion & Purification Kit (Roboklon). Primer sequences were designed to incorporate SfiI recognition sequences at both sides of each oligonucleotide. The resulting PCR products were size-selected using Agencourt AMPure XP beads (Beckman Coulter).

**Promoter-Proximal MPRA empty plasmid backbone construction**

The empty plasmid backbone for the MPRA plasmid pool was designed using pGL4.23[luc2/minP] (Promega) as a template. The pGL4.23[luc2/minP] backbone was PCR amplified using PfuUltra II Fusion HS DNA Polymerase (Agilent) with a phosphorylated primer targeting immediately downstream of the XbaI site (downstream of the luc2 gene) and a phosphorylated primer targeting the HindIII site (upstream of the minimal promoter). The resulting PCR product was self-ligated using Quick T4 DNA Ligase (NEB) and transformed into One Shot MAX Efficiency DH5alpha-T1R Competent Cells (Life Technologies). The empty plasmid backbone was isolated using the QIAGEN Plasmid Plus Maxi Kit (Qiagen).

**Promoter-Proximal MPRA plasmid pool cloning**

The MPRA empty plasmid backbone and the oligonucleotide library were digested with SfiI (NEB). The digested backbone was purified using the QIAquick PCR Purification Kit (Qiagen) and the digested oligonucleotide library was isolated using the MinElute PCR Purification Kit (Qiagen). The resulting digested products were ligated overnight with concentrated T4 DNA Ligase (NEB). The entire ligation reaction was transformed into One Shot MAX Efficiency DH5alpha-T1R Competent Cells (Life Technologies) and the resulting MPRA plasmid pool (Promoter-Proximal) was isolated using the QIAGEN Plasmid Plus Maxi Kit (Qiagen).

**Promoter-Proximal MPRA reporter gene insert cloning**

The MPRA reporter gene insert was designed to contain a minimal promoter upstream of a GFP reporter gene. The minimal promoter was PCR amplified from pGL4.23[luc2/minP] (Promega) using PfuUltra II Fusion HS DNA Polymerase (Agilent) and primers designed to incorporate an upstream KpnI recognition sequence and a downstream AgeI recognition sequence. The resulting PCR product was triple digested with KpnI-HF (NEB), AgeI-HF (NEB), and DpnI (NEB). The GFP reporter gene was PCR amplified from peTurboGFP-dest1 (Evrogen) using PfuUltra II Fusion HS DNA Polymerase (Agilent) and primers designed to incorporate an upstream AgeI recognition sequence and a downstream XbaI recognition sequence. The resulting PCR product was triple digested with AgeI-HF (NEB), XbaI (NEB), and DpnI (NEB). Digested PCR products were isolated using the QIAquick PCR Purification Kit (Qiagen). To generate a plasmid backbone for cloning, pGL4.23[luc2/minP] (Promega) was double digested with KpnI-HF (NEB) and XbaI (NEB) followed by gel purification using the QIAquick Gel Extraction Kit (Qiagen). Digested PCR products and the plasmid backbone were ligated using Quick T4 DNA Ligase (NEB), transformed into One Shot MAX Efficiency DH5alpha-T1R Competent Cells (Life Technologies), and isolated using the QIAGEN Plasmid Plus Maxi Kit (Qiagen). The resulting plasmids were double digested with KpnI (NEB) and XbaI (NEB) followed by gel purification of the MPRA reporter gene insert (minimal promoter and GFP) using the QIAquick Gel Extraction Kit (Qiagen).

**Promoter-Proximal MPRA plasmid pool + reporter gene insert cloning**

The MPRA plasmid pool (Promoter-Proximal) was double digested with KpnI-HF (NEB) and XbaI (NEB) followed by gel purification using the QIAquick Gel Extraction Kit (Qiagen). The digested plasmid pool and the previously digested reporter insert were ligated overnight with concentrated T4 DNA Ligase (NEB). The entire ligation reaction was transformed into One Shot MAX Efficiency DH5alpha-T1R Competent Cells (Life Technologies) and the final MPRA plasmid pool + reporter gene insert (Promoter-Proximal) was isolated using the QIAGEN Plasmid Plus Maxi Kit (Qiagen).

**Promoter-Distal MPRA plasmid cloning**

The Promoter-Distal MPRA vector was designed using the Promoter-Proximal MPRA vector as a template. The empty plasmid backbone from the Promoter-Proximal MPRA was first modified to remove all AgeI and NotI recognition sequences from the vector. To generate the GFP reporter gene, the minimal promoter and GFP were PCR amplified from the Promoter-Proximal MPRA vector using PfuUltra II Fusion HS DNA Polymerase (Agilent) and primers designed to incorporate an upstream SfiI recognition sequence and downstream AgeI, NotI, and SfiI recognition sequences. The modified MPRA empty plasmid backbone and GFP reporter gene were digested with SfiI (NEB). The digested backbone and reporter gene were purified using the QIAquick PCR Purification Kit (Qiagen). The resulting digested products were ligated using Quick T4 DNA Ligase (NEB) and transformed into One Shot MAX Efficiency DH5alpha-T1R Competent Cells (Life Technologies). The empty Promoter-Distal MPRA vector was isolated using the QIAGEN Plasmid Plus Maxi Kit (Qiagen). After observing very low reporter gene expression in pilot Promoter-Distal MPRA experiments the minimal promoter was replaced with the SV40 promoter in the empty Promoter-Distal MPRA vector.

**Promoter-Distal Oligonucleotide library amplification**

The oligonucleotide library was amplified by PCR with Q5 High-Fidelity DNA Polymerase (NEB) and primers targeting the universal primer sequences within the oligonucleotides. To preserve library complexity PCR was performed using the Micellula DNA Emulsion & Purification Kit (Roboklon). Primer sequences were designed to incorporate upstream AgeI and downstream NotI recognition sequences into each oligonucleotide. The resulting PCR products were size-selected using Agencourt AMPure XP beads (Beckman Coulter).

**Promoter-Distal MPRA plasmid pool cloning**

The empty Promoter-Distal MPRA vector and the oligonucleotide library were double digested with AgeI-HF (NEB) and NotI-HF (NEB). The digested MPRA vector was purified using the QIAquick PCR Purification Kit (Qiagen) and the digested oligonucleotide library was isolated using the MinElute PCR Purification Kit (Qiagen). The resulting digested products were ligated overnight with concentrated T4 DNA Ligase (NEB). The entire ligation reaction was transformed into One Shot MAX Efficiency DH5alpha-T1R Competent Cells (Life Technologies) and the resulting MPRA plasmid pool (Promoter-Distal) was isolated using the QIAGEN Plasmid Plus Maxi Kit (Qiagen).
