# Supplementary figures and images for: p53 regulates enhancer accessibility and activity in response to DNA damage
Source: Nucleic Acids Res. 2017 Jul 13;45(17):9889–900. doi: 10.1093/nar/gkx577 (PMC5622327; doi:10.1093/nar/gkx577)

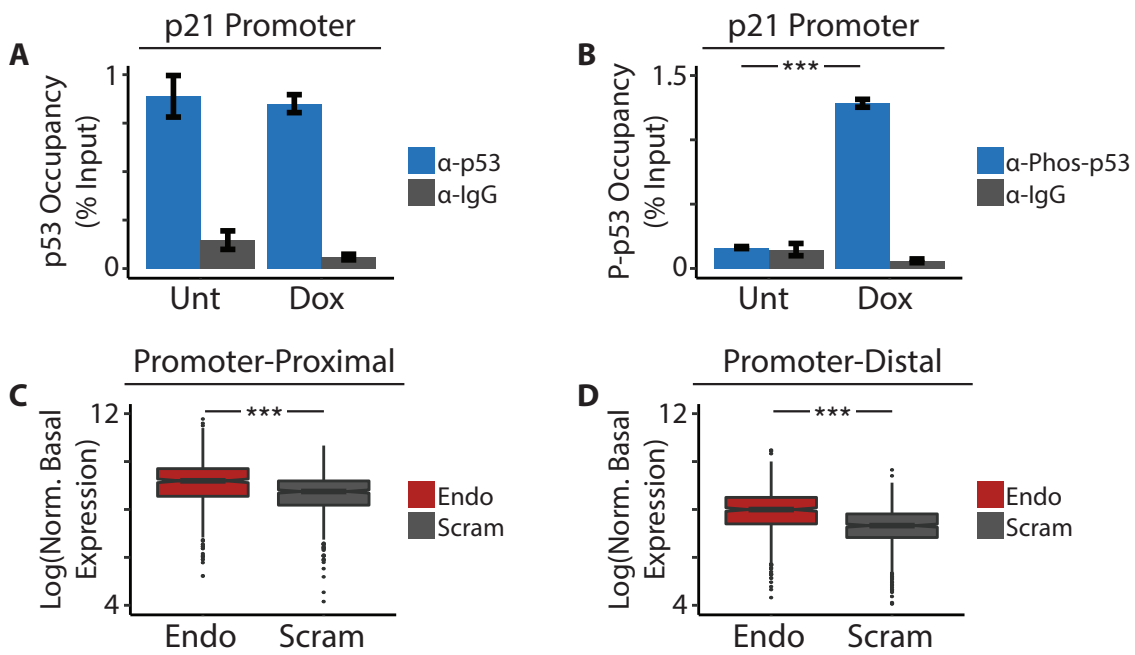

Supplement: Supplementary Data [file gkx577_supp.zip › FigureSUP1.pdf]

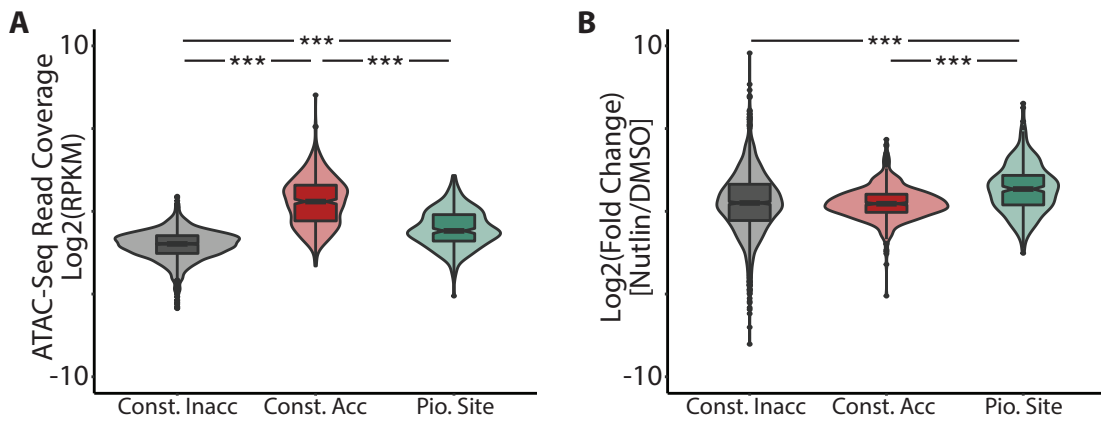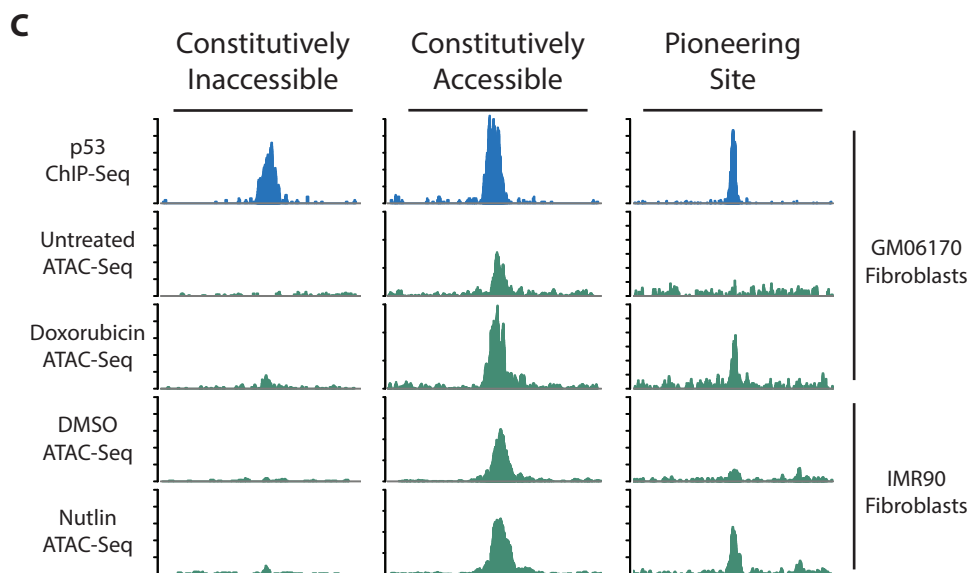

Supplement: Supplementary Data [file gkx577_supp.zip › FigureSUP2.pdf]

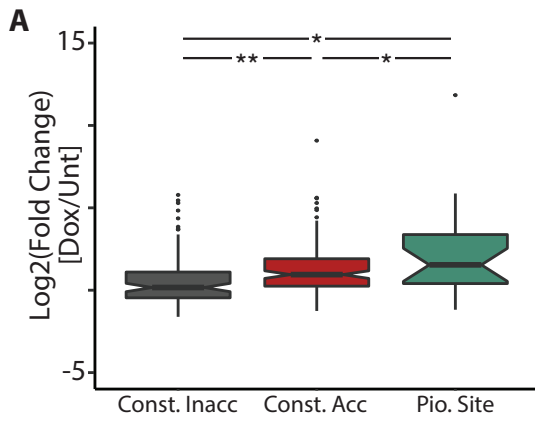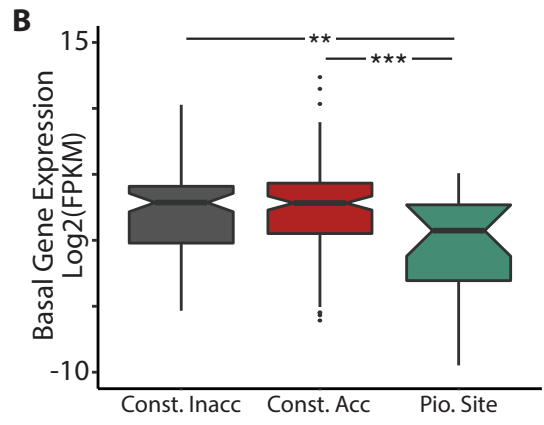

Supplement: Supplementary Data [file gkx577_supp.zip › FigureSUP3.pdf]

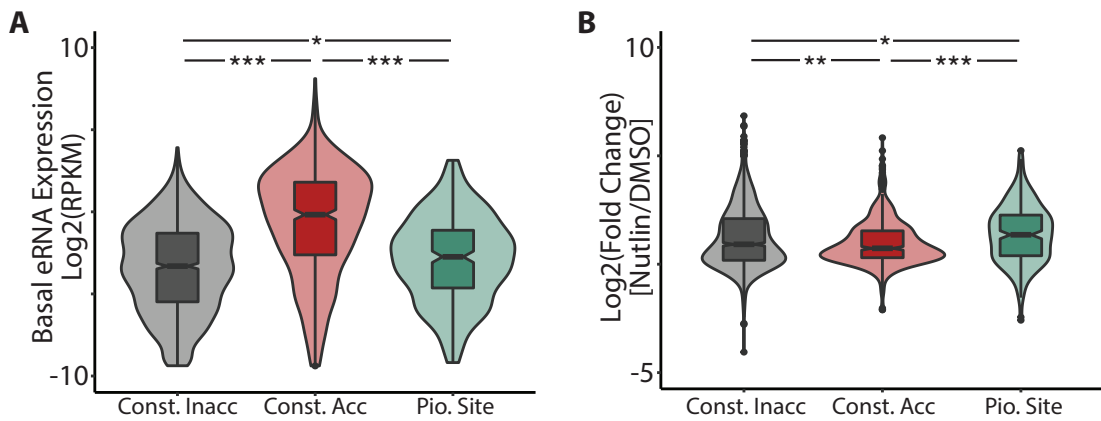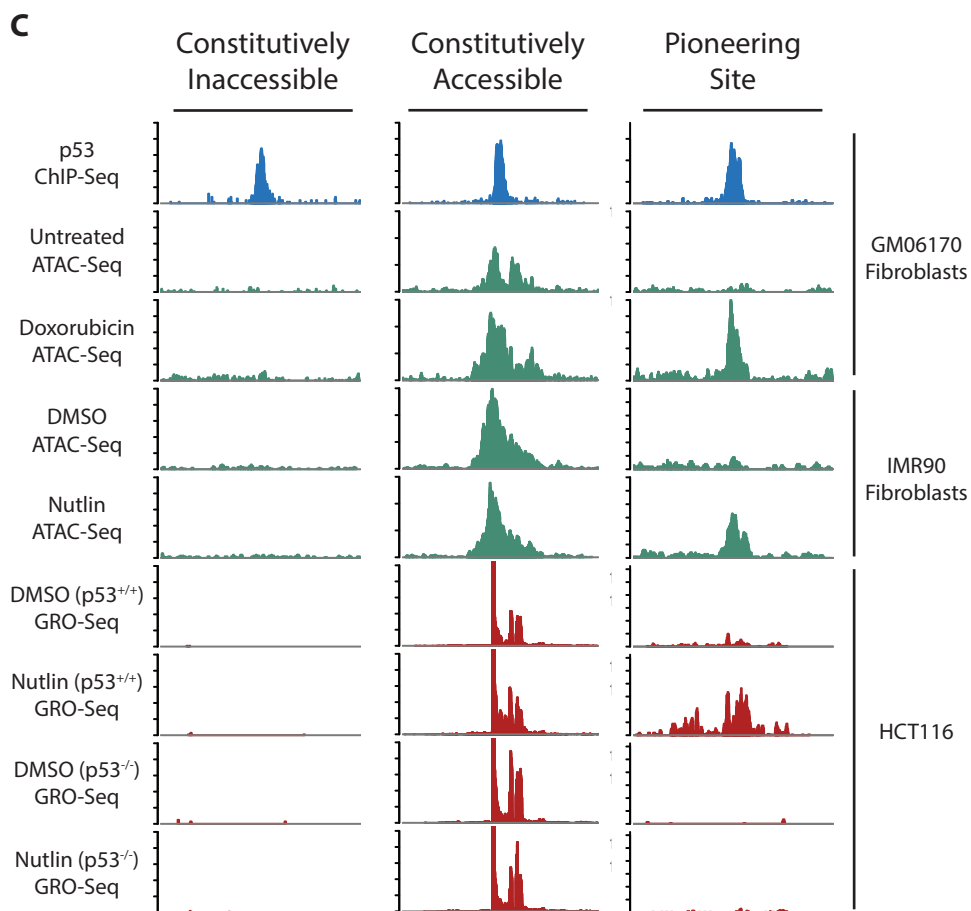

Supplement: Supplementary Data [file gkx577_supp.zip › FigureSUP4.pdf]

**A**

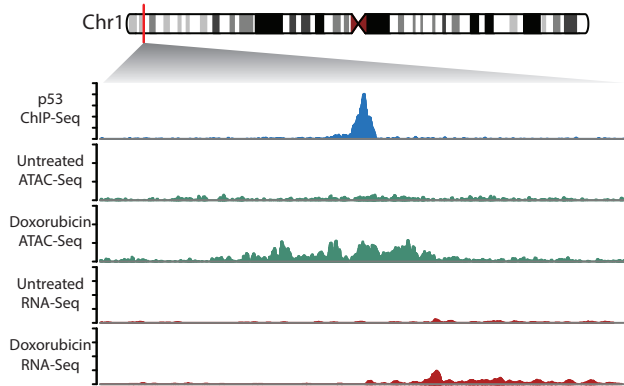

**B**

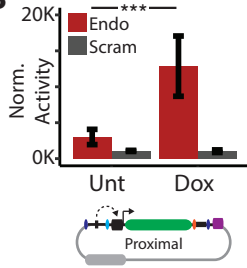

**C**

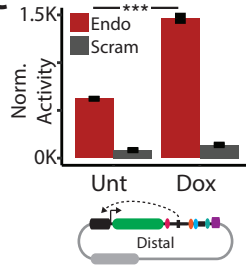

**D**

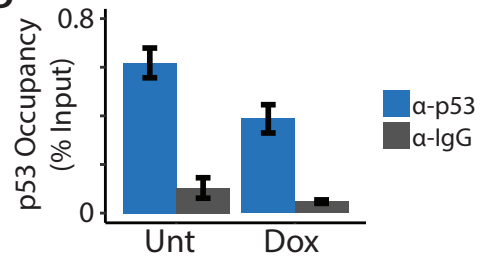

**E**

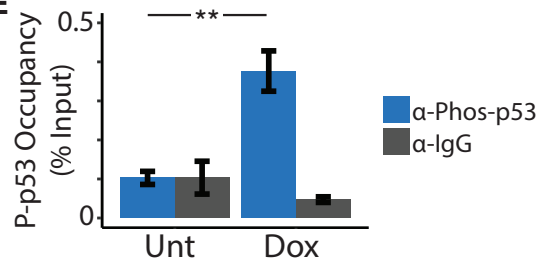

Supplement: Supplementary Data [file gkx577_supp.zip › FigureSUP5.pdf]
